# Investigating the impact of parallel media engagement initiatives on suicide reporting in Canada and Israel

**DOI:** 10.1007/s00127-025-02886-4

**Published:** 2025-04-07

**Authors:** Mark Sinyor, Daniella Ekstein, Prudence Po Ming Chan, Yu Vera Men, Racheli Starostintzki Malonek, Ayal Schaffer, Thomas Niederkrotenthaler, Marnin J. Heisel, Benjamin I. Goldstein, Donald A. Redelmeier, Paul Taylor, Rachel Mitchell, Rosalie Steinberg, Yossi Levi-Belz

**Affiliations:** 1 Department of Psychiatry, Sunnybrook Health Sciences Centre, Toronto, Canada; 2 Department of Psychiatry, University of Toronto, Toronto, Canada; 3 The Lior Tsfaty Center for Suicide and Mental Pain Studies, Ruppin Academic Center, Emek Hefer, 40250 Israel; 4 Department of Social and Preventive Medicine, Centre for Public Health, Unit Public Mental Health Research, Medical University of Vienna, Vienna, Austria; 5 Wiener Werkstaette for Suicide Research, Vienna, Austria; 6 Department of Psychiatry, The University of Western Ontario, London, Canada; 7 Centre for Youth Bipolar Disorder, Center for Addiction and Mental Health, Toronto, Canada; 8 Institute for Clinical Evaluative Sciences, Toronto, Canada; 9 Department of Medicine, University of Toronto, Toronto, Canada; 10 Division of General Internal Medicine, Sunnybrook Health Sciences Centre, Toronto, Canada; 11 Sunnybrook Health Sciences Centre, Toronto, Canada

**Keywords:** Suicide, Media reporting, Media guidelines, Cross-national, Canada, Israel

## Abstract

**Purpose:**

To contrast changes in suicide-related media reporting quality during parallel initiatives to engage national media in Canada and Israel.

**Methods:**

We coded media articles in Canada’s and Israel’s highest circulating newspapers (major broadsheet and tabloid newspapers, respectively) for putatively harmful and putatively protective suicide-related content. A sample of 150 articles (30/year) from each country was randomly selected for three time points: 2012 (T1; prior to media engagement), 2016–2017 (T2; early media engagement), and 2018–2019 (T3; late media engagement). Chi-square tests and binary logistic regression investigated overall between-country differences in reporting quality over time.

**Results:**

Following media engagement, adherence to guidelines improved over time in both countries for most variables. Over time, fewer Canadian and more Israeli articles covered celebrity suicide (OR = 4.97; 95%CI 1.68–16.69); more Canadian and fewer Israeli articles covered warning signs for suicide (OR = 0.30; 95%CI 0.12–0.78). Comparing articles over the entire timespan (T1-T3), a higher proportion of Israeli tabloid articles included putatively harmful content, such as mentioning suicide means (Israel: 65.3% vs. Canada 25.3%, χ^2^(1) = 48.4, *p* < 0.001), and a higher proportion of Canadian broadsheet articles included putatively protective content, such as providing information on intervention (Israel: 2.0% vs. Canada 27.3%, χ^2^(1) = 38.5, *p* < 0.001).

**Conclusion:**

Media engagement appeared to confer benefits in both countries and publication formats. A higher proportion of Canadian articles adhered to several specific recommendations. Our findings must be interpreted in the context of differences in format between major Canadian and Israeli newspapers (broadsheet vs. tabloid) and the much higher total volume of suicide-related articles in Canada.

**Supplementary Information:**

The online version contains supplementary material available at 10.1007/s00127-025-02886-4.

## Introduction

Engagement with media to promote responsible suicide-related reporting has been identified by the World Health Organization as one of four key evidence-based strategies for suicide prevention at the population level (the other three being means restriction, life skills for youth and early identification and support) [1]. This recommendation arises based on increasingly strong evidence that certain kinds of reporting, specifically stories focused on the death of identifiable individuals and/or ones that model how to die by suicide, are often associated with increased subsequent suicides [2–5], whereas stories of survival and help-seeking are often associated with fewer subsequent suicides [2, 6–9].

Over the past decade, suicide prevention experts in many countries have engaged with media on suicide reporting as a public health prevention strategy; [10–29] however journalist awareness of responsible media guidelines and adherence to them remains variable and is low in many countries worldwide [30–37]. Dissemination of media guidelines may be conceptualized as an intervention designed to improve reporting; guidelines are often integrated within broader initiatives aimed at engaging with journalists and other content creators both in formal and informal settings to promote responsible suicide-related reporting. In Canada, in 2014–2015, members of the suicide prevention community together with journalists launched a dialogue to improve the quality of suicide-related reporting in the popular press; these efforts culminated in the release of jointly-authored Canadian Psychiatric Association Guidelines for media reporting on suicide in early 2018 [38–40]. In addition to guidelines, these efforts, which we described in detail previously [38], encompassed regular dialogue with journalists, meetings, symposia, and presentations over the course of four years (2015–2018; heretofore referred to as the “Canadian initiative”). Similarly, in 2014, Israel launched its National Suicide Prevention Program (NSPP); one of the main aims of which was to educate both the public and journalists about preventing suicide [41]. This effort (heretofore referred to as the “Israeli initiative”) involved recommendations for responsible reporting that were disseminated more passively than in the Canadian initiative as well as awareness campaigns for the public that are believed to have also influenced the tone and content of subsequent newspaper coverage [41]. Note that, while there can be nuanced differences in guidelines between countries, there is relatively strong agreement internationally on elements of reporting that are recommended and discouraged and therefore the specific guidance provided to Canadian and Israeli journalists was similar.

Our research groups have each conducted similar studies of the impact of these efforts on adherence to responsible reporting recommendations in our respective presses. The confluence of intervention dates and data collection periods creates a unique opportunity to compare findings between countries. In Canada, engagement with the media was associated with reductions in the proportion of articles published containing several putatively harmful characteristics (e.g. harmful imagery and mentions of a suicide method) but also some increases in concerning content (e.g. simplistic reasons for suicide) [40]. Engagement was also associated with increases in putatively protective content, although with the caveats that the absolute amount of this content remained somewhat low and that the overarching narratives of most articles remained problematic from a safe messaging perspective [40]. In Israel, the NSPP was likewise associated with reductions in putatively harmful content following its launch [41]. However, as in Canada, the results were somewhat mixed, as there was no observed improvement in the proportion of articles with putatively protective content after the NSPP [41]. Interestingly, improvements in guideline adherence observed just after the release of NSPP appeared to diminish slightly in the subsequent two years (2018–2019). Notably, one key difference between the two countries that emerges in examining our prior respective studies is that there is a far higher volume of suicide reporting in Canada compared to Israel [40, 41].

To our knowledge, there have been no studies to date examining between-country differences in the impact of simultaneous media engagement initiatives encouraging responsible reporting about suicide. This may partly arise because media initiatives and guidelines are often country-specific, but likely also because the timing and focus of initiatives and data available on their potential impact may be quite variable. The similar timing and aims of media initiatives in Canada and Israel creates a unique natural experiment, and the availability of similar data from our prior studies enables us to contrast outcomes between Canada and Israel. Studies of this kind are necessary because the number of national initiatives to engage media worldwide is increasing, yet there is limited information about the differences between those initiatives and whether their impact on adherence to responsible media guidelines varies. This study is an initial effort to fill that gap.

For context, media markets in Canada and Israel share some similarities but also differences. In Canada, the General Social Survey in 2013 (near the outset of the epoch for our study) showed that Canadians are generally following news and current affairs less frequently than in the past [42]. Compared to younger Canadians (15–24 years old), older Canadians (aged 55 and older) were more likely to follow current affairs daily, more likely to read newspapers, magazines and watch television for an average of eight hours per week [42]. Canadians with a higher level of education were also more inclined to follow news and current affairs regularly. The most popular mode of media to follow news and current affairs in 2013 was television (78%), followed by Internet (59%), newspaper (51%), radio (50%) and magazines (17%) [32]. A majority of those (77%) who used the Internet to follow news and current affairs were 15–34 years old, compared to 36% for those 55 and older [42].

In Israel, there is strong cross-ownership across different sources of media like television, radio, printed press and the internet for news [43] The most widely read newspaper is “Israel Hayom”, followed closely by “Yediot Ahronoth”, though showing a steady decline in exposure from 2014 to 2018 [44]. As in Canada, the majority of Israelis consume news through television with somewhat fewer using news websites (42%) [45]. Israelis also have high rates of social media use with many using social media to access news [46].

As our prior work has already demonstrated improved reporting over time in both countries, we thus focus this study on differences in overall reporting quality between them and on the magnitude of improvement following media engagement. It is important for us to note at the outset one key difference between the countries and data. In Canada, the most widely circulated newspapers across the country are in broadsheet format whereas in Israel the most widely circulated newspapers are tabloids. For this reason, the previous Canadian study collected data from broadsheet newspapers and the Israeli study examined tabloids. This study will therefore focus on the content of media reporting that the population is primarily exposed to in Israel and Canada. Given this difference in format, we anticipate that the proportion of articles adhering to responsible media reporting guidelines will be higher in the Canadian broadsheet sample than the Israeli tabloid sample.

## Methods

### Exposure of interest and data sources

#### Media engagement initiatives

The exposures of interest in this study are the media engagement initiatives in each country. Implementation of the Canadian initiative commenced in 2015 and, between 2015 and 2018, involved two national fora for journalists, a presentation at the annual national journalism conference, two symposia at a national psychiatric conference that journalists participated in, and publication of updated responsible reporting guidelines that were contributed to and disseminated by journalists [40]. In Israel, following onset of the NSPP in 2014, there were several workshops with journalists and editors with the main newspapers in Israel, as well as publications of updated responsible suicide-reporting guidelines, which were delivered to all main newspapers in the country. Moreover, on the initiation of the Israeli initiative, there were several remarks by its leaders regarding the importance of responsible reporting on suicide events and its consequences regarding diminishing the suicide rate.

#### Newspapers and article selection

Our outcome of interest was the quality (as measured by adherence to responsible reporting guidelines) [39, 47] of suicide-related reporting in print and online articles published in major national newspapers in Canada (Globe & Mail, Toronto Star, National Post) and Israel (Yediot Ahronot [translation:“Latest News”] and Israel Hayom [translation: “Israel Today”]). Note again that the two sets of newspapers differ in that the Canadian newspapers are published in broadsheet format and the Israeli newspapers in tabloid format. Broadsheet newspapers have a more traditional and formal newspaper format that more commonly involves in-depth analysis, whereas tabloids often have a smaller amount of text, shorter stories, more sensationalistic headlines, and more images.

Our research groups identified and coded suicide-related articles from the above newspapers according to previously published methods [40–41, 48]. The Israeli study focused exclusively on three time periods (T1-2012; T2-2016-2017 and T3 2018–2019) and, therefore, to ensure an analogous comparison, we only used Canadian data from the same years. Note that the Canadian studies involved single independent coding of each article by trained reviewers following establishment of inter-rater reliability. All variables included in this study had κ > 0.8 with the exception of warning signs for suicide (κ = 0.74) and simplistic reasons for suicide (κ = 0.73) [48]. The Israeli study involved coding of each article by multiple trained independent reviewers. Using a random sample of 20% of analyzed articles in the Israeli study, the proportion of agreement for variables ranged from 0.79 to 0.94 [41].

Reviewing the two databases, we were able to match eleven putatively harmful article characteristics coded in both studies (the word “suicide” appearing in the headline, any suicide method mentioned, method (hanging), method (firearm), method (jumping), method (drowning), method (other), celebrity suicide, glorified presentation of suicide, simplistic reasons for suicide, potentially harmful images) and three putatively protective article characteristics (warning signs for suicide, how to intervene, other prevention information).

After reviewing the counts of available articles from studies in both countries, we identified that it would be feasible to select 30 articles per year for each country for the years in which comparator data were available (2012, 2016–2019). Our goal was to include a balanced number of articles from the included publications. Given that there were three Canadian publications and two Israeli publications with the largest circulation in each country, we aimed for 10 and 15 articles per year from each country’s newspapers respectively. If there were that number or fewer articles from a specific newspaper in any year, all were selected for inclusion. Moreover, if there were excess articles coded for a specific year, a random number generator was used to select articles to arrive at the total of 30 per year from each country.

### Statistical analysis

We used chi square tests to investigate overall differences in reporting quality across all years between the two countries. We also conducted a sensitivity analysis to determine if the results differed for only time-points T2 and T3 (i.e. after the media interventions began). In order to identify independent differences in reporting quality between the two countries, binary logistic regressions were performed on all articles using country, year and country by year interaction; a stratification analysis was also conducted within the country strata controlling across all time-points. Outcome variables were the word “suicide” appearing in the headline, any suicide method mentioned, method (hanging), method (firearm), method (jumping), method (drowning), method (other), celebrity suicide, glorified presentation of suicide, simplistic reasons for suicide, potentially harmful images) and three putatively protective article characteristics (warning signs for suicide, how to intervene, other prevention information). Adjusted odds ratio (aORs) (95% confidence interval [95% CI]) were reported. All analyses were conducted using SPSS Version 28.0.1.1. Given the lack of prior related findings, we opted not to adjust for multiple tests and maintained a significance level of 0.05.

## Results

Of the 300 total articles identified for inclusion, 54 were from the Toronto Star, 53 were from the National Post, 43 were from the Globe and Mail (Canada) and there were 75 each from Yediot Ahronot and Israel Hayom (Israel). Overall differences between the two countries in measures of reporting quality across all years of study are presented in Table 1.

**Table 1:** Putatively harmful and protective characteristics of articles focusing on suicide in Canadian broadsheet newspapers (*n* = 150) and Israeli tabloid newspapers (*n* = 150) during the years of matching data availability (2012, 2016–2019)

Characteristics of media item	Canada (*n*, %)	Israel (*n*, %)	χ2	df	*p*
**Putatively harmful**					
Word “suicide” in the headline	4 (2.7)	17 (11.3)	9.46	1	0.002
Any Method	38 (25.3)	98 (65.3)	48.4	1	< 0.001
Method (hanging)	10 (6.7)	34 (22.7)	15.34	1	< 0.001
Method (firearm)	14 (9.3)	32 (21.3)	8.32	1	0.004
Method (jumping)	6 (4.0)	15 (10.0)	4.15	1	0.04
Method (drowning)	0 (0.0)	3 (2.0)	3.03	1	0.08
Method (other)	9 (6.0)	14 (9.3)	1.18	1	0.28
Celebrity suicide	12 (8%)	44 (29.3)	22.76	1	< 0.001
Simplistic reasons for suicide	42 (28.0)	94 (62.7)	36.37	1	< 0.001
Glorifying suicide^a^	1 (0.7)	50 (33.3)	73.15	1	< 0.001
Potentially harmful images	4 (2.7)	24 (16.0)	15.76	1	< 0.001
**Putatively protective**					
Warning signs for suicide	21 (14.0)	30 (20.0)	1.91	1	0.17
How to intervene	41 (27.3)	3 (2.0)	38.546	**1**	< 0.001
Prevention information	22 (14.6)	10 (6.7)	5.04	1	0.03

^a^Only 120 Israeli articles were coded for this variable as it was added midway through this study

**Fig. 1:**
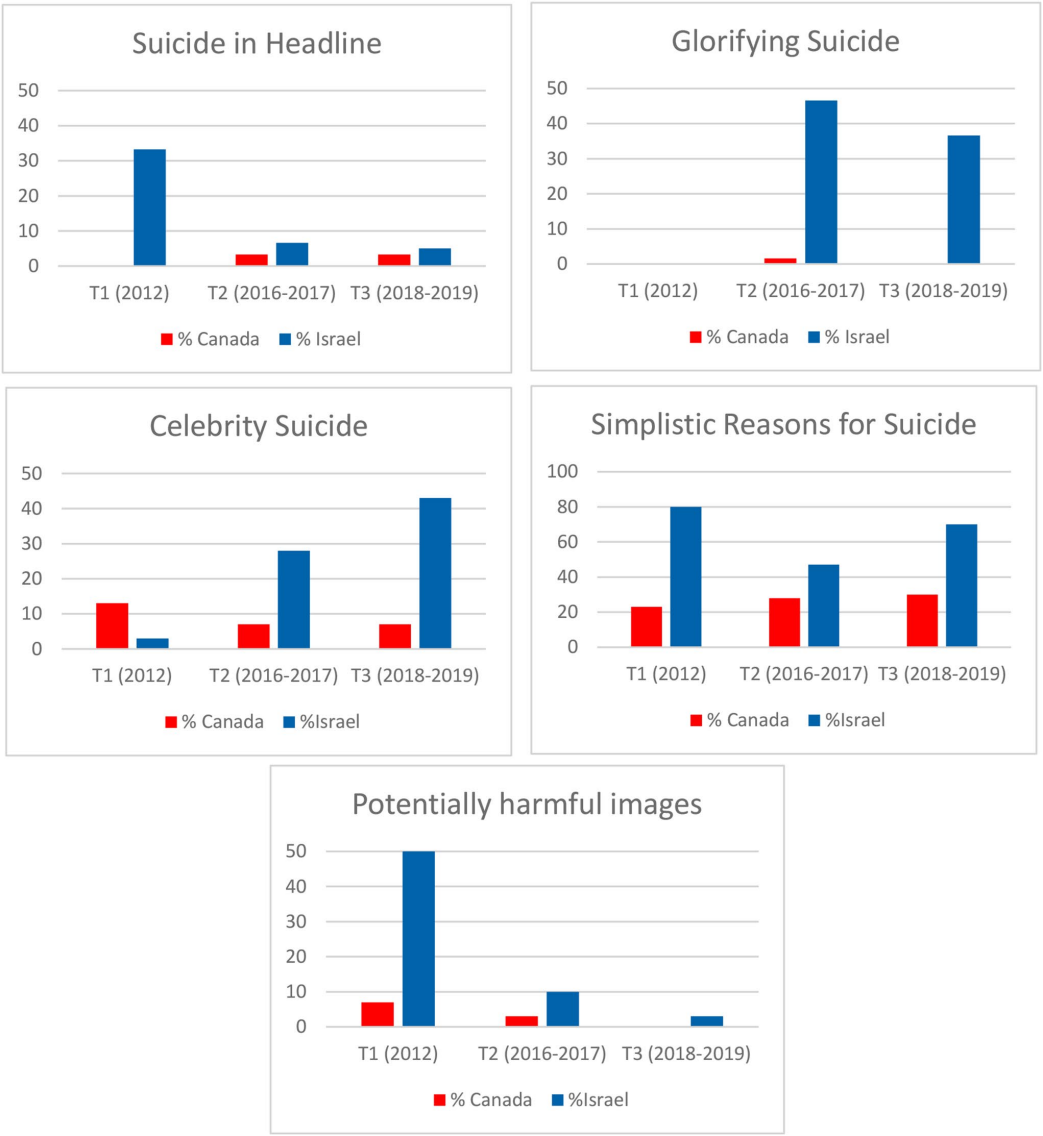
Proportion of Suicide-Related Articles with Putatively Harmful Characteristics (excluding suicide methods) in Canadian Broadsheet Newspapers (*n* = 150) and Israeli Tabloid Newspapers (*n* = 150) During the Three Study Epochs (2012, 2016–2017, 2018–2019)

**Fig. 2:**
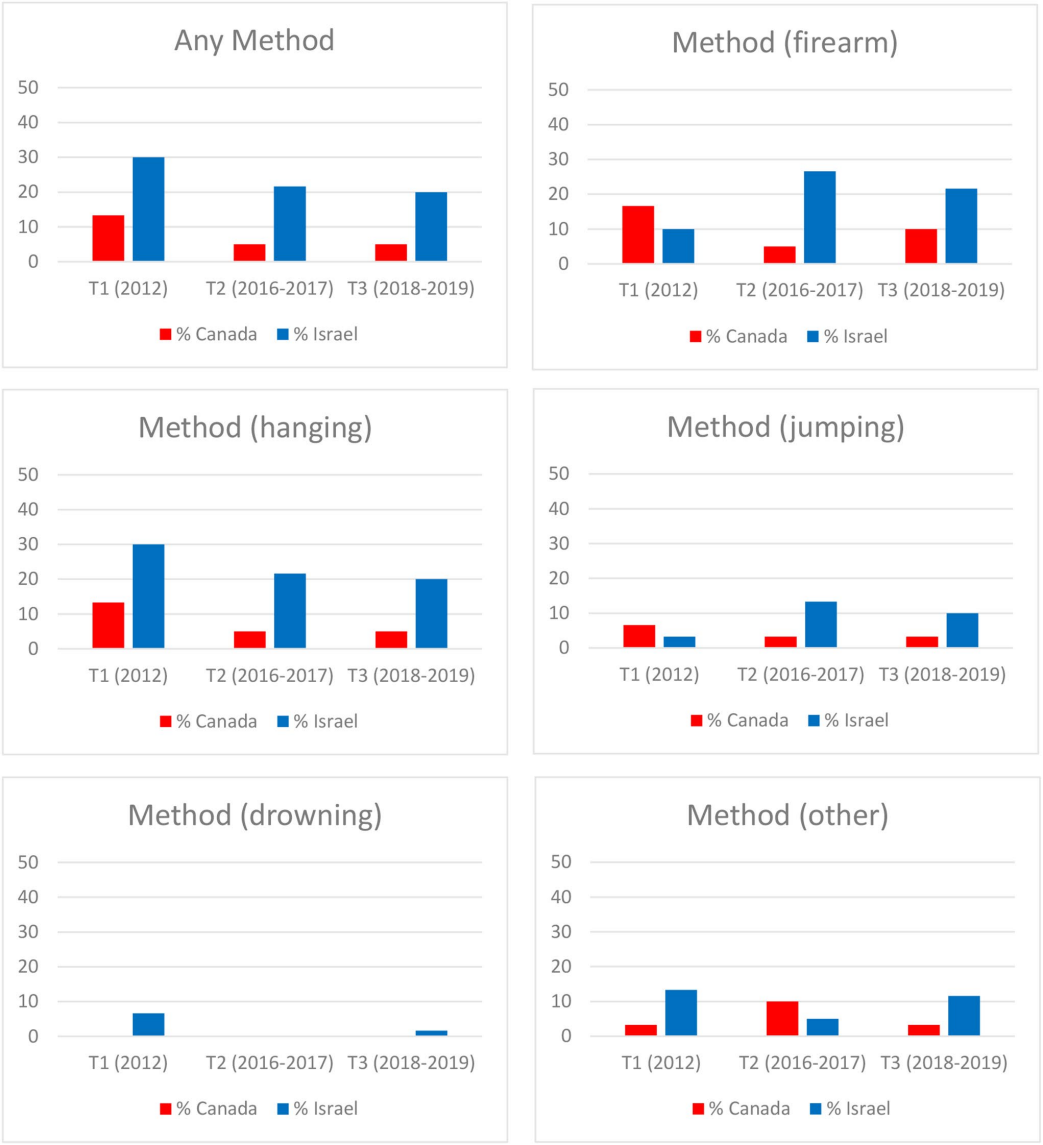
Proportion of Suicide-Related Articles with Suicide Methods in Canadian Broadsheet Newspapers (*n* = 150) and Israeli Tabloid Newspapers (*n* = 150) During the Three Study Epochs (2012, 2016–2017, 2018–2019)

**Fig. 3:**
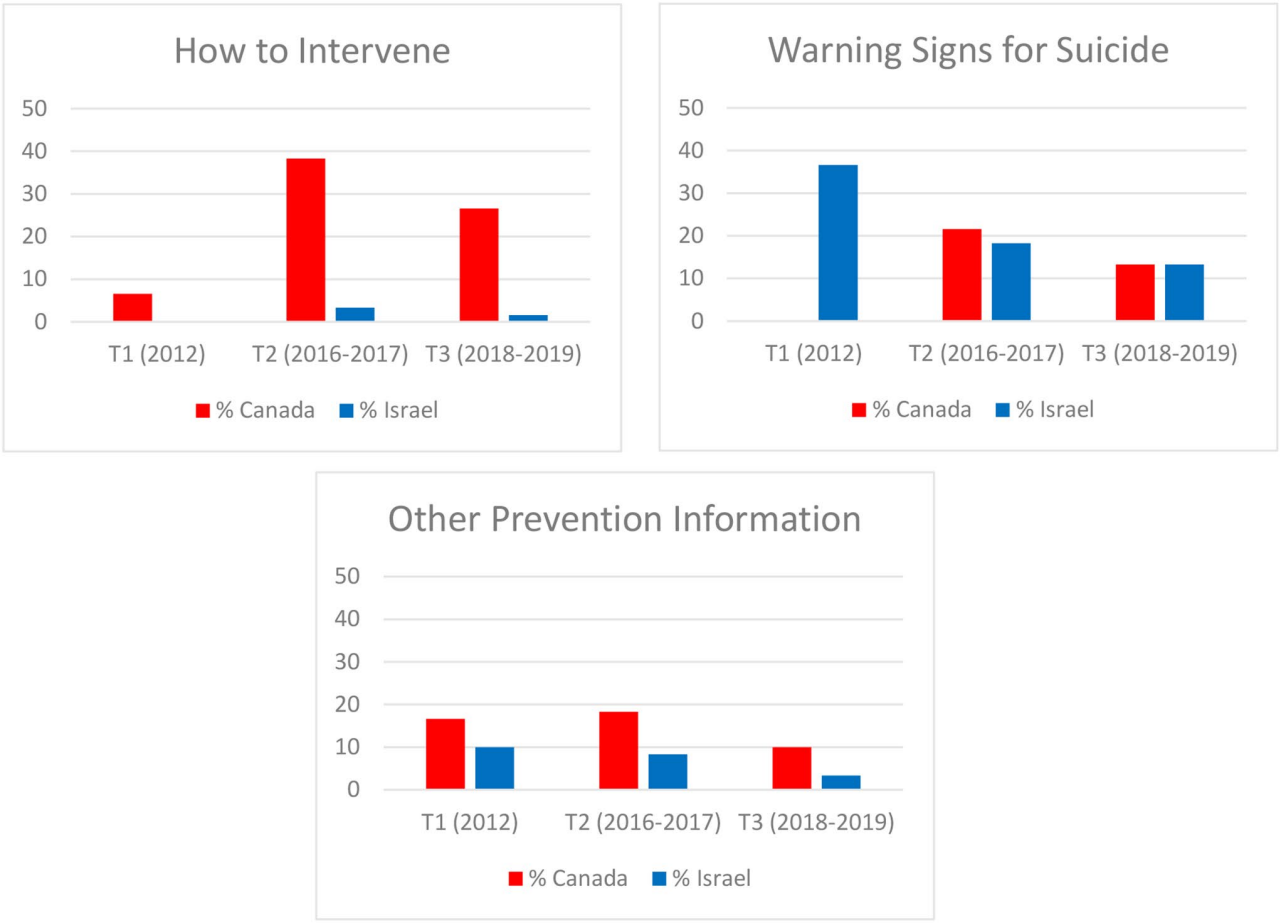
Proportion of Suicide-Related Articles with Putatively Protective Characteristics in Canadian Broadsheet Newspapers (*n* = 150) and Israeli Broadsheet Newspapers (*n* = 150) During the Three Study Epochs (2012, 2016–2017, 2018–2019)

Changes in the proportion of articles with putatively harmful and protective factors across T1-T3 are presented in Figs. 1, 2 and 3. The quality of reporting appeared to improve over time in both countries with respect to most variables. The notable outlier was articles glorifying suicide in Israel which were absent in 2012 but increased substantially at the later time points.

Most variables differed between countries and, in each case, Israeli tabloid articles had a higher proportion of articles with putatively harmful content and Canadian broadsheet articles had a higher proportion of articles with putatively protective content. The largest differences with respect to putatively harmful content were for any method mentioned (Israel: 65% vs. Canada 25%, χ^2^(1) = 48.4, *p* < 0.001), method (hanging) (Israel: 22.7% vs. Canada 6.7%, χ^2^(1) = 15.3, *p* < 0.001), celebrity suicide (Israel: 29% vs. Canada 8%, χ^2^(1) = 22.8, *p* < 0.001), simplistic reasons for suicide method (hanging) (Israel: 63% vs. Canada 29%, χ^2^(1) = 36.4, *p* < 0.001), glorifying suicide (Israel: 33% vs. Canada 1%, χ^2^(1) = 73.1, *p* < 0.001), and potentially harmful images (Israel: 16% vs. Canada 3%, χ^2^(1) = 15.8, *p* < 0.001). The largest difference for putatively protective content was for information about how to intervene (Israel: 2% vs. Canada 27%, χ^2^(1) = 38.5, *p* < 0.001).

Results of our sensitivity analysis which focused exclusively on the years following commencement of the media engagement initiatives (2016–2019) were nearly identical to the overall results (Table 2). Only one variable produced different results– there was no longer a difference in the proportion of articles with the word “suicide” in the headline (Israel: 6% vs. Canada 3%, χ2(1) = 0.88, *p* = 0.35).

**Table 2:** Putatively harmful and protective characteristics of articles focusing on suicide in Canadian broadsheet newspapers (*n* = 120) and Israeli tabloid newspapers (*n* = 120) after commencement of media engagement efforts (2016–2019)

Characteristics of media item	Canada (*n*, %)	Israel (*n*, %)	χ2	df	*p*
**Putatively harmful**					
Word “suicide” in the headline	4 (3.3)	7 (5.9)^a^	0.884	1	0.35
Any Method	26 (21.7)	79 (65.8)	47.560	1	< 0.001
Method (hanging)	6 (5.0)	25 (20.8)	13.472	1	< 0.001
Method (firearm)	9 (7.5)	29 (24.2)	12.507	1	< 0.001
Method (jumping)	4 (3.3)	14 (11.7)	6.006	1	0.01
Method (drowning)	0 (0.0)	1 (0.8)	1.004	1	0.32
Method (other)	8 (6.7)	10 (8.3)	0.240	1	0.62
Celebrity suicide	8 (6.7)	43 (35.8)	30.501	1	< 0.001
Simplistic reasons for suicide	35 (29.2)	70 (58.3)	20.741	1	< 0.001
Glorifying suicide	1 (0.8)	50 (41.7)	59.782	1	< 0.001
Potentially harmful images	2 (1.7)	8 (6.7)	3.757	1	0.05
**Putatively protective**					
Warning signs for suicide	21 (17.5)	19 (15.8)	0.120	1	0.73
How to intervene	39 (32.5)	3 (2.5)	37.403	1	< 0.001
Prevention information	17 (14.2)	7 (5.8)	4.630	1	0.03

Binary logistic regression results are presented in Supplementary Tables 1 and 2. These were consistent with the results described above and identified a relationship between time and country for two specific article characteristics. The country-by-year interaction term revealed significant relationships between celebrity suicides and warning signs for suicide. Specifically, a smaller proportion of Canadian articles reported on celebrity suicide over the time points, whereas the proportion grew in Israeli articles (OR = 4.97; 95%CI 1.68–16.69; *p* = 0.004). Also, a larger proportion of Canadian articles reported on warning signs for suicide over time with the opposite trend observed for Israeli articles (OR = 0.30; 95%CI 0.12–0.78; *p* = 0.01).

## Discussion

In this study, we presented the results of a natural experiment involving initiatives to improve media reporting in Canada and Israel. Such efforts are ongoing in many countries worldwide; however, the confluence of initiative dates and data collection in these two countries allowed us to make specific comparisons, albeit with caveats, most importantly the difference in format between their most circulated newspapers (broadsheet in Canada vs. tabloid in Israel). We confirmed the finding of improvements in reporting quality over time in both countries. This is highly encouraging as our study lends some support to the notion that interventions to promote responsible media reporting appear to impact both broadsheet and tabloid reporting. Given that the tabloid format used in major newspapers in Israel generally results in more sensational news stories discouraged by media guidelines, we expected to find differences in overall reporting quality between the countries and indeed did find differences. A higher proportion of the Canadian broadsheet articles adhered to reporting guidelines throughout the course of the study period than the Israeli tabloids. We also specifically found a smaller proportion of articles about celebrities and a greater proportion highlighting warning signs in the Canadian articles over time with the reverse trend in the Israeli articles.

There are different ways to interpret these findings. The obvious one is that broadsheet reporting is expected to adhere to higher journalistic standards than tabloid reporting and that this accounts for the observed differences. The finding that between-country differences were already apparent in T1 (i.e. prior to media engagement) suggests that this explanation likely accounts for at least some of the findings. It would be important for future, prospective, studies to aim to compare newspapers with the same format across countries. Another possibility is that, over the past decade, Canadian journalists have had greater awareness of and/or receptivity towards responsible suicide-related reporting and/or that media outreach efforts in Canada have been more impactful. If the latter is true, it may owe to the fact that there was frequent ongoing dialogue between suicide messaging experts and the Canadian media after 2015 [40] may have been a more robust intervention than the Israeli initiative, which involved early-phase collaborations with, and media campaigns for, journalists. No formal measurements were made about the number of journalists exposed to the initiatives in each country. However, we will note anecdotally, that a substantial majority of the major press in Canada would have had some awareness of and exposure to the Canadian initiative whereas uptake in Israel may have been weaker.

It is also logical to expect some differences in reporting between countries. Canada and Israel are similar in that they are both industrialized, democratic countries, with robust public health policies and a free press. They differ in many other respects, for example, in terms of culture, religion, language, and world region. These latter factors which are complex and are not amenable to a simple summary likely also played a role. It is also important to emphasize that the countries differ substantial in suicide rates; in 2019, Canada had a rate of 10.3 per 100,000 per year whereas Israel had half that rate (5.2 per 100,000 per year) [49].

However, this way of considering the results merely addresses a narrow question: if a journalist publishes an article in the most circulated newspaper in a specific country, what are the chances that it will include certain types of potentially harmful and/or helpful information? A key question from a public health standpoint is the degree to which the public is exposed to harmful and/or helpful content. To address this, we must consider both proportions of articles with different characteristics and their absolute number. Even though the proportion of articles with putatively harmful elements in the Canadian broadsheet media was smaller than that observed in articles published in Israel tabloids, the absolute proportions of articles with such content in Canada (e.g., 25% describing a suicide method) is of substantial concern. The earlier study which examined all articles on suicide in the two Israeli tabloid newspapers found that the combined yearly total number of articles ranged from 30 to 51 [41]. Contrast that with the most recent Canadian data showing that, across two years (2020–2021), five major news outlets in Canada, including the three we used for this study, published 3,065 suicide related articles [40]. Taking our finding that 65% of articles in Israel mention a suicide method compared to 25% in Canada, a reader of one of the Israeli newspapers would be expected to encounter 10–15 articles per year with that content whereas a reader of one of the Canadian newspapers would be expected to encounter 10-times more or approximately 150 such articles. These figures suggest that although Canadian major newspapers have a broadsheet format and would be expected to adhere to higher reporting standards, the Canadian public are nevertheless exposed to a far larger absolute number of articles with harmful content than the Israeli public do through their tabloids simply due to the volume of reports. Note that the same would be true for protective content. Such an interpretation must also be considered in the context of Canada having a much larger population than Israel with an accordingly larger number of media outlets.

The results must also be considered in the context of recent findings by one of our groups that, whereas the Canadian media is performing better in recent years on a checklist of article quality characteristics such as those included in this study, the large majority of articles continue to have overarching narratives that are potentially harmful [40]. Although, to our knowledge, no studies have examined article overarching narratives in the Israeli press, our findings here, including the high proportion of articles in T2 and T3 with content glorifying suicide [41], provide a strong indication that we would likely observe similar narrative results in Israel. Our results must also be considered in relation to country-specific historical findings. For example, an older Israeli study found that suicide reporting dramatically increased between the 1950s and 1980/90s despite relatively consistent suicide rates [50]. A narrative comparison of historical practices of suicide-related reporting in Canada and in Israel found that, in both countries, journalists previously avoided reporting on suicide for ethical reasons but that these norms loosened to different degrees over time [51].

Taken together, we would suggest that our results emphasize that (a) as identified by studies in other countries [52, 53], initiatives to improve media reporting can result in meaningful change in the quality of reporting across countries and reporting format (as was observed in both Canada and Israel), (b) there are notable and relevant between-country differences in the way that the media reports on suicide with implications for prevention, and (c) clearly substantial work is still needed in both Canada and Israel to ensure that the public receives appropriate information and messages about suicide conducive to its prevention. In particular, there is a need to work with the tabloid media in Israel to increase the proportion of articles that are adherent to guidelines. There is also a need to address the high volume of suicide reporting in Canada that collectively exposes the public to large quantities of content that violates media guidelines. Such efforts must account for the ever-changing media landscape and, in particular, the shift towards consumption of news through social media. Engagement must therefore go beyond journalists and traditional media sources.

This study has a number of limitations. The most important limitation is the difference in newspaper type (broadsheet vs. tabloid) which is a potential confounder relevant in interpreting between-country differences. Because of our study design, the comparisons in this paper should be understood less as comparisons of analogous reporting types, but comparisons between the *main media exposures occurring in the two countries*. That is, we examined the most circulated newspapers to which each population was exposed and not the entire media landscape. Findings may have been different across the latter especially if we had compared newspapers of the same type between countries. Notably, our results do indicate that media initiatives appear to have helped improve reporting in both countries– i.e. both for broadsheet and tabloid formats. In future, prospective studies should collect data from media sources with the same format. We also relied on a sample of articles from five specific publications and five specific years and cannot determine the degree to which that sample may or may not be representative of articles in other publications or published in other years. Reliance only on newspaper reporting is also a key limitation given that many people in both countries are likely to consume news in different formats (e.g. television); we nevertheless were reliant on the data available from prior studies by our groups which focused only on newspapers. We did not directly measure the impact of the media engagement strategies in Canada or Israel and therefore cannot draw conclusions about whether and to what degree change over time was influenced by those strategies or other factors. Knowledge of and beliefs regarding the importance of responsible reporting of suicide likely varies between journalists in both countries and this study was not designed to identify or characterize such differences. This study was also not designed to examine which sorts of suicide stories received coverage in each country and the degree to which coverage of stories was in alignment with each nation’s prevalence of suicide. Our analysis was also limited by both the number of newspapers and articles available in the two parent studies from which the current study arose and ideally future studies would include larger samples both in terms of news outlets and articles. As described above, this study did not examine overarching narratives related to suicide which may differ in important ways from the specific characteristics of articles. The study also focused on two countries over a specific epoch and each with specific media initiatives. Results may have differed in other countries, epochs, and with different initiatives. Canada and Israel are two of dozens of countries worldwide that have created initiatives aimed at improving suicide-related reporting. Ideally, future studies would identify other groups of countries that spearhead similar initiatives at similar times, collect comparable data, and run further comparisons to determine whether the results we observed are consistent across other cultures and regions. Such studies should also aim to examine the impact of changes in reporting on deaths by suicide. We were also bound by variables previously abstracted for both countries, allowing for comparisons, and cannot comment on whether results would have differed for other putatively harmful and/or protective content. Development of our coding scheme and variables also relied on media guidelines that were available during the epoch of study. The recommendations in guidelines have evolved over time and further research should explore the impact of updated guidance for journalists. Lastly, our study was not designed to examine any potential changes over time with respect to the quality of social media content, another key media exposure relevant to suicide prevention.

To our knowledge, this is the first study to examine differences in suicide-related reporting quality between countries in the context of parallel media outreach initiatives. It demonstrates that cross-national comparisons can be informative and, given the increasing globalization of both news stories and media, we would argue that scientists should strongly consider including multiple nations in studies of suicide and the media. The results demonstrate that, among those articles selected for analyses in the current study, guideline adherence was higher in major Canadian broadsheet newspapers than in major Israeli tabloids, although this must be understood in the context of a media environment in Canada that has a far higher volume of suicide-related coverage. The differences we observed likely arise due to the combination of differences in newspaper format and culture, although further research is needed to confirm that speculation. Nevertheless, the results reaffirm that efforts to engage with journalists appear to be having an impact but that there is still substantial room for improvement in both countries. Future studies in these and other countries should explore the quality of reporting as it evolves over time with special attention to those putatively protective aspects of reporting that can save lives.

## Electronic supplementary material

Below is the link to the electronic supplementary material.

Supplementary Material 1

## Data Availability

Data is available as publicly available media reports.
