# Supplementary material for: Investigating the impact of parallel media engagement initiatives on suicide reporting in Canada and Israel
Source: Soc Psychiatry Psychiatr Epidemiol. 2025 Apr 7;60(8):1871–82. doi: 10.1007/s00127-025-02886-4 (PMC12325504; doi:10.1007/s00127-025-02886-4)
Supplement: Supplementary file 1 — Supplementary Material 1 [file 127_2025_2886_MOESM1_ESM.docx]

Table 1. Binary logistic regression for putatively harmful and protective characteristics while controlling for country and time periods

|  | Adjusted odds ratios (95% confidence intervals) | | | | | | | | | | | | |
| --- | --- | --- | --- | --- | --- | --- | --- | --- | --- | --- | --- | --- | --- |
|  | **Putatively harmful** | | | | | | | | | | **Putatively protective** | | |
|  | Word “suicide” in the headline | Any method reported | Method (hanging) | Method (firearm) | Method (jumping) | Method (other) | Celebrity suicide | Simplistic reasons for suicide | Glorifying suicide | Potentially harmful images | Warning signs for suicide | How to intervene | Prevention information |
| **Country (Canada as reference)** | Reference | Reference | Reference | Reference | Reference | Reference | Reference | Reference | Reference | Reference | Reference | Reference | Reference |
| Israel | **238.733(4.80-11878.34)** | 2.25 (0.50-10.20) | 2.42 (0.31-18.61) | 0.83 (0.10-6.79) | 0.51 (0.02-13.15) | 1.07 (0.08-14.63) | 0.12 (0.01-1.49) | **7.01 (1.51-32.63)** | NA | **22.25 (1.39-356.34)** | **20.37 (2.33-178.41)** | 0.05 (0.00-9.56) | 0.74 (0.07-7.68) |
| **Time period (2012 as reference)** | Reference | Reference | Reference | Reference | Reference | Reference | Reference | Reference | Reference | Reference | Reference | Reference | Reference |
| T2 (2016-2017) | 5.31 (0.19-148.39) | 0.38 (0.12-1.23) | 0.34 (0.06-2.05) | 0.52 (0.10-2.84) | 0.51 (0.04-6.75) | 0.66 (0.08-5.43) | 0.20 (0.03-1.18) | 0.70 (0.22-2.25) | NA | 0.40 (0.02-9.05) | **8.42 (1.26-56.10)** | 8.89 (0.57-139.43) | 1.49 (0.24-9.12) |
| T3 (2018-2019) | 94.82 (0.28-32705.63) | 0.19 (0.02-1.58) | 0.20 (0.01-5.91) | 0.21 (0.01-5.03) | 0.11 (0.00-16.18) | 0.49 (0.01-21.91) | **0.02 (0.00-0.69)** | 1.71 (0.22-13.37) | NA | 0.27 (0.00-116.15) | **31.14 (1.29-754.02)** | 4.87 (0.03-707.28) | 1.00 (0.04-23.32) |
| **Interaction term** | 0.16 (0.03-0.80) | 1.52 (0.78-2.96) | 1.31 (0.50-3.40) | 1.70 (0.67-4.28) | 2.14 (0.50-9.24) | 1.21 (0.39-3.80) | **4.97 (1.68-14.69)** | 0.82 (0.42-1.57) | NA | 0.55 (0.11-2.85) | **0.30 (0.12-0.78)** | 1.05 (0.11-9.73) | 0.75 (0.24-2.30) |

NA = Not applicable. Category has missing values and therefore logistic regression cannot be ra

|  | Adjusted odds ratios (95% confidence intervals) | | | | | | | | | | | | |
| --- | --- | --- | --- | --- | --- | --- | --- | --- | --- | --- | --- | --- | --- |
|  | **Putatively harmful** | | | | | | | | | | **Putatively protective** | | |
|  | Word “suicide” in the headline | Any method reported | Method (hanging) | Method (firearm) | Method (jumping) | Method (other) | Celebrity suicide | Simplistic reasons for suicide | Glorifying suicide | Potentially harmful images | Warning signs for suicide | How to intervene | Prevention information |
| **Canada** | | | | | | | | | | | | | |
| **Time period (2012 as reference)** | Reference | Reference | Reference | Reference | Reference | Reference | Reference | Reference | Reference | Reference | Reference | Reference | Reference |
| T2 (2016-2017) | NA | 0.42 (0.16-1.08) | 0.34 (0.07-1.64) | 0.26 (0.06-1.19) | 0.48 (0.07-3.61) | 3.22 (0.37-28.07) | 0.46 (0.11-2.00) | 1.30 (0.47-3.59) | NA | 0.48 (0.07-3.61) | NA | 8.70 (1.89-40.04) | 1.12 (0.35-3.59) |
| T3 (2018-2019) | NA | 0.42 (0.16-1.08) | 0.34 (0.07-1.64) | 0.56 (0.16-1.99) | 0.48 (0.07-3.61) | 1.00 (0.09-11.49) | 0.46 (0.11-2.00) | 1.41 (0.51-3.87) | NA | NA | NA | 5.09 (1.09-23.85) | 0.56 (0.16-1.99) |
| **Israel** | | | | | | | | | | | | | |
| **Time period (2012 as reference)** | Reference | Reference | Reference | Reference | Reference | Reference | Reference | Reference | Reference | Reference | Reference | Reference | Reference |
| T2 (2016-2017) | **0.10 (0.03-0.35)** | 1.16 (0.46-2.90) | 0.65 (0.24-1.74) | 3.27 (0.87-12.29) | 4.45 (0.53-37.47) | 0.34 (0.07-1.64) | **11.47 (1.45-90.95)** | **0.22 (0.08-0.61)** | 0.66 (0.32-1.37) | **0.10 (0.03-0.29)** | 0.39 (0.14-1.04) | NA | 0.82 (0.18-3.68) |
| T3 (2018-2019) | **0.07 (0.02-0.28)** | 1.08 (0.43-2.68) | 0.58 (0.21-1.59) | 2.49 (0.65-9.52) | 3.22 (0.37-28.07) | 0.86 (0.23-3.20) | **22.18 (2.83-173.62)** | 0.58 (0.20-1.67) | NA | **0.03 (0.01-0.15)** | **0.27 (0.09-0.76)** | NA | 0.31 (0.05-1.97) |

Table 2. Binary logistic regression - stratification analysis for putatively harmful and protective characteristics by country
